# HIV, Syphilis, and Behavioral Risk Factors among Female Sex Workers before and after Implementation of Harm Reduction Programs in a High Drug-Using Area of China

**DOI:** 10.1371/journal.pone.0084950

**Published:** 2014-01-08

**Authors:** Li Zhang, Shu Liang, Weixia Lu, Stephen W. Pan, Benli Song, Qianping Liu, Yunan Xu, Hui Dong, Hui Xing, Yiming Shao, Yuhua Ruan

**Affiliations:** 1 Beijing Centers for Disease Control and Prevention, Beijing, China; 2 Sichuan Provincial Center for Disease Control and Prevention, Chengdu, Sichuan, China; 3 State Key Laboratory for Infectious Disease Prevention and Control, National Center for AIDS/STD Control and Prevention, Chinese Center for Disease Control and Prevention, Collaborative Innovation Center for Diagnosis and Treatment of Infectious Diseases, Beijing, China; 4 The School of Population and Public Health University of British Columbia, Vancouver, British Columbia, Canada; 5 Xichang Center for STD and Leprosy Control, Xichang City, Sichuan, China; 6 Beijing Youth Politics College, Beijing, China; George Mason University, United States of America

## Abstract

**Objective:**

To evaluate the impact of harm reduction programs on HIV and syphilis infection and related risk behaviors among female sex workers (FSWs) in a drug trafficking city in Southwest China.

**Design:**

Before and after harm reduction program study.

**Methods:**

Two cross-sectional surveys were conducted among FSWs before and after harm reduction programs were launched in Xichang city, Sichuan province. The first and second cross-sectional surveys were conducted in 2004 and 2010, respectively. Temporal changes in odds of HIV, syphilis, and behavioral risk factors were assessed by multivariable logistic regression while controlling for socio-demographics.

**Results:**

The 2004 and 2010 cross-sectional surveys recruited 343 and 404 FSWs, respectively. From 2004 to 2010, the odds of syphilis infection decreased by 35% and was of borderline statistical significance (AOR: 0.65, 95% CI: 0.41–1.03), while odds of HIV infection rose, but not significantly (AOR: 4.12, 95% CI: 0.76–22.45). Although odds of unprotected sex with primary sex partners did not significantly change over time (AOR: 0.96; 95% CI: 0.61–1.50), odds of unprotected sex with clients declined significantly and remarkably (AOR: 0.14, 95% CI: 0.09–0.21). Notably, the odds of reporting ≥10 new sex partners in the previous month increased by 37% (AOR: 1.37; 95% CI: 0.98–1.90).

**Conclusions:**

Harm reduction strategies may be an effective means of reducing unprotected sex with clients among FSWs. Future research is needed to better target both FSWs and IDUs and interrupt bridging networks for HIV transmission in high drug-using areas of China.

## Introduction

Prior to 1949, syphilis was one of the most common sexually transmitted infections (STI) in China [1]–[3]. In response, Chairman Mao launched a nationwide STI control campaign throughout the 1950s that entailed shutting down brothels, screening and treatment of syphilis, and mass media education and prevention [4]–[5]. By 1964, syphilis infections were virtually eliminated [4]–[5]. However, since its adoption of free market economic policies in 1978, China has witnessed the rapid reemergence of global drug trafficking, abuse and addiction, commercial sex, and STIs. Due to rapid economic development, large income disparities, limited occupational opportunities for women, and changing notions of sexuality, it is estimated that the number of commercial sex workers nationwide increased 140-fold, from 25,000 to 3.5 million sex workers between the years 1985 and 2000 [6]–[7]. During this same period, the annual reported cases of eight notified sexually transmitted diseases (STDs, including syphilis and HIV/AIDS) increased by 147-fold from 5,838 to 859,040 cases [8]. In recent years, syphilis and HIV accounted for over 10% of all reported STDs cases nationwide [9]–[13].

China is no exception to the evolution of the HIV epidemic occurring in other Asian countries: beginning as a drug-driven epidemic and shifting to one driven predominantly by sexually contact [7]. National HIV molecular surveys in China indicate that most cases of unknown transmission have similar HIV clade profiles as sexually transmitted cases [14]. In 2012, 68% of reported HIV/AIDS cases were believe to be infected by heterosexual transmission [15]. Heterosexual transmission of HIV through female sex workers (FSWs) is of particular concern [7]. National sentinel surveillance data revealed that HIV prevalence among FSWs rose from 0.02 percent in 1996 to 0.93 percent in 2004 [16], before peaking in 1999 (1.5%) [17]. Since the 2000s, HIV prevalence among FSWs has remained relatively low and stable, ranging from 0.0% to 10.3% (median, 0.6%); corresponding prevalence rates for active syphilis range from 0.8% to 12.5% (median, 6.9%) [17]. Herpes, chlamydia, gonorrhea, and trichomoniasis are also commonly reported STIs among Chinese FSWs [17].

China's government has led an ambitious and comprehensive response to the national HIV/AIDS epidemic. The centerpiece of China's National AIDS Control Policy has been the “Four Frees and One Care” program in 2003, which was designed to provide free voluntary counseling and testing, free anti-viral treatment, free prevention of mother to child transmission (PMTCT), free education to AIDS orphans, and financial assistance and social support to HIV/AIDS patients [18]. As part of the “Four Frees and One Care” initiative, harm reduction programs such as knowledge education, HIV testing and counselling, condom promotion, methadone maintenance therapy and needle exchange were rapidly scaled-up and implemented at the national and local levels in 2004 [18]–[21]. The goal of the present study is to evaluate the impact of the “Four Frees and One Care” harm reduction programs on HIV and syphilis infection and related risk behaviors among female sex workers (FSWs) in a drug trafficking city in Southwest China.

## Methods

### Study setting

This study was conducted in Xichang City, Sichuan Province of China, a city which lies along one of China's major drug trafficking routes to northwest, south, and central China from the “Golden Triangle” in Southeast Asia, one of the world's largest hubs for the illicit production and distribution of heroin [22]–[25]. The total population of Xichang is about 600,000, among which 10% are of the ethnic minority Yi group, and 100,000 are migrants to the city. Between 2004 and 2010, the estimated total number of female sex workers in Xichang decreased from 2800 to 2000, while the corresponding number of drug users decreased from 3200 to 2500.

In 2004, Xichang city began implementation of the following harm reduction programs: HIV/AIDS knowledge education, HIV testing and counselling, condom promotion, methadone maintenance therapy and needle exchange [18]–[21], [25], [26]. HIV testing and counselling, condom promotion and prevention interventions were managed by local healthcare providers. Specifically, condom promotion entailed the provision of free condoms and educational materials at healthcare provider clinics and entertainment establishments. Between 2004 and 2010, public health intervention outreach staff conducted monthly field visits to all known entertainment establishments (n>100) to distribute free condoms and safe sex literature. Peer FSWs also made weekly visits to FSWs working in beauty salons and on the street in order to distribute free condoms and literature about safe sex practices. Meetings with peer FSWs were convened each three months in order to update study staff about progress and to receive ongoing training about promoting skills in safer sex. In total, 38172 condoms and 2623 informational flyers were distributed to 1864 FSW.

### Study design and study population

Participants of two cross-sectional studies (cross-sectional study 2004 and cross-sectional study 2010) were enrolled in 2004 and 2010. Recruitment was primarily driven by community-based outreach after FSW-associated venues in Xichang city were systematically mapped out by experienced outreach staff, who then conducted field visits to FSW-associated venues to distribute study-related information to FSWs and invite FSW to voluntarily participate in the study. Outreach staff also invited brothel managers and FSWs to refer other FSWs to the study. Participant eligibility criteria included female, self-report of commercial sex in the past 6 months, and able and willing to provide written informed consent. Additional details of the study protocol and design have been described elsewhere [27]. The study protocol and informed consent were approved by the Institutional Review Board of the Chinese Center for AIDS/STD Control and Prevention. All potential participants provide their written informed consent to participate in this study.

### Data collection

Each study participant was assigned a unique identifier code in order to ensure confidentiality. An interviewer-administrated questionnaire was used to collect data on demographic characteristics (age, gender, ethnicity, education, previous employment, current marital status, income, birth area and current residence) and behavioral information (age at sexual debut, age when first engaged in commercial sex, establishment of sex work, frequency of sexual intercourse with primary sex partners and commercial sex clients in the past 6 months, condom use in the last month, number of new sex partners in the past 6 months, average fee collected per sexual act, and drug use in the past 3 months).

### Laboratory tests

A whole blood sample was collected from each participant in order to test for HIV and syphilis antibodies. HIV infection status was determined by an enzyme immunoassay (EIA) (Beijing Wantai Biological Medicine Company, China) and an HIV-1/2 Western Blot confirmation (HIV Blot 2.2 WB™, Genelabs Diagnostics). Syphilis infection was determined using an EIA (Beijing Jinhao Biological Production Company, Beijing, China) and confirmed using a Treponemal Pallidum Particle Agglutination (TPPA) test for detection of antibodies to *Treponema pallidum* (TPPA™, FUJIREBIO, Japan).

### Data analysis

Original questionnaires and laboratory testing data were double entered and validated with EpiData software (EpiData 3.0 for Windows; The EpiData Association Odense, Denmark). After cleaning, the data were then transferred into a SAS database for analysis (SAS 9.1 for Windows; SAS Institute Inc., NC, USA). Univariate proportions and means were calculated for categorical and continuous variables, respectively. To assess crude differences between the 2004 and 2010 cross-sectional studies, chi-square tests and t-tests were used for categorical and continuous variables, respectively. Multivariate logistic regression was used to assess temporal changes in the odds of HIV and syphilis infection and related behavioral variables, while controlling for age, ethnicity, years of education, previous occupation, marital/cohabitation status, annual income and birth area.

## Results

The 2004 and 2010 cross-sectional surveys successfully enrolled 343 and 404 FSWs, respectively. The numbers of participants who refused to participate or dropped out of the study were 28 and 8 FSW for the 2004 and 2010 cross-sectional surveys, respectively. Table 1 presents participant characteristics of the two independent cross-sectional surveys, as well as crude P-values to assess the significance of differences. The average ages and standard deviations of study participants in the 2004 and 2010 surveys were 24±5 and 26±6 years, respectively. Ethnic minorities represented 12.0% of the 2004 survey and 18.3% of the 2010 survey. The proportion of participants who engaged in farming prior to sex work decreased from 34.1% in 2004 to 19.8% in 2010. More FSWs (72.9%) in the 2004 survey were married or cohabiting compared to those of the 2010 survey (59.7%). Significant differences between the 2004 and 2010 surveys were found for age, ethnicity, previous occupation, marital/cohabitation status, annual income, and birth area (P<0.05).

**Table 1:** Sociodemographic characteristics of participants in cross-sectional study 2004 and cross-sectional study 2010.

Factors	Cross-sectional study 2004 N (%)	Cross-sectional study 2010 N (%)	P *-*value
Total	343	404	
Age (mean ± SD, year)	24±5	26±6	<0.03
Ethnicity			
Han majority	302(88.0)	330(81.7)	
Yi and other minorities	41(12.0)	74(18.3)	0.02
Years of education			
≤6	111(32.4)	142(35.1)	
>6	232(67.6)	262(64.9)	0.42
Previous occupation			
Non-farmers	226(65.9)	324(80.2)	
Farmers	117(34.1)	80(19.8)	<0.0001
Currently married or cohabiting			
No	93(27.1)	163(40.3)	
Yes	250(72.9)	241(59.7)	<0.001
Yearly income (US $)			
<3258	243(70.8)	173(42.8)	
≥3258	100(29.2)	231(57.2)	<0.0001
Birth area			
Rural	241(70.3)	316(78.2)	
Urban	102(29.7)	88(21.8)	0.013
Local Xichang resident			
No	287(83.7)	337(83.4)	
Yes	56(16.3)	67(16.6)	0.92

Compared to participants of the 2004 survey, 2010 survey participants were more likely to have begun sex work after age 21 (60.6% vs. 53.1%, P = 0.04), more likely to have been involved in sex work for at least two years (43.3% vs. 35.3%, P = 0.03), less likely to have a primary sex partner in the last six months (45.0% vs. 59.2%, P<0.0001), more likely to report at least ten acts of commercial sex in the last month (79.2% vs. 70.8, P = 0.01), less likely to report unprotected sex with clients in the last month (9.4% vs. 44.3%, P<0.0001), more likely to report at least ten new sex partners in the past month (67.1% vs. 57.4%, P = 0.01), less likely to report drug use in the last three months (1.7% vs. 7.0%, P<0.001), and more likely to have received HIV testing (43.8% vs. 19.5%, P<0.0001). Between 2004 and 2010, prevalence of HIV rose from 0.6% to 1.5% (P = 0.25), while prevalence of syphilis decreased from 15.7% to 12.1% (P = 0.15). Neither change was statistically significant (Table 2).

**Table 2:** Behavioral characteristics and HIV and Syphilis status of participants in cross-sectional study 2004 and cross-sectional study 2010.

Factors	Cross-sectional study 2004 N (%)	Cross-sectional study 2010 N (%)	P *-*value
Total	343	404	
Place of sex work			
High-end	174(50.7)	208(51.5)	
Low-end	169(49.3)	196(48.5)	0.84
Average charge per sex trade (US $)			
<16.3	115(33.5)	89(22.0)	
≥16.3	228(66.5)	315(78.0)	<0.001
Age at sex debut (years)			
<18	124(36.2)	148(36.6)	
≥18	219(63.8)	256(63.4)	0.89
Age of initiating sex work (years)			
<21	161(46.9)	159(39.4)	
≥21	182(53.1)	245(60.6)	0.04
Duration of sex work (years)			
<2	222(64.7)	229(56.7)	
≥2	121(35.3)	175(43.3)	0.03
Having primary sex partner(s) in the last 6 months			
No	140(40.8)	222(55.0)	
Yes	203(59.2)	182(45.0)	<0.001
Having unprotected sex with primary sex partner(s) in the last month			
No	282(82.2)	318(78.7)	
Yes	61(17.8)	86(21.3)	0.23
Number of sex trades in the last month			
<10	100(29.2)	84(20.8)	
≥10	243(70.8)	320(79.2)	0.01
Having unprotected sex with clients in the last month			
No	191(55.7)	366(90.6)	
Yes	152(44.3)	38(9.4)	<0.0001
Number of new sex partner(s) in the last 1 months≥10			
No	146(42.6)	133(32.9)	
Yes	197(57.4)	271(67.1)	0.01
Used drug in the last 3 months			
No	319(93.0)	397(98.3)	
Yes	24(7.0)	7(1.7)	<0.001
Utilized MMT in the last 3 months			
No	330(96.2)	404(100.0)	
Yes	13(3.8)	0(0.0)	0.93
Received HIV testing			
No	276(80.5)	227(56.2)	
Yes	67(19.5)	177(43.8)	<0.0001
Syphilis(+)			
No	289(84.3)	355(87.9)	
Yes	54(15.7)	49(12.1)	0.15
HIV(+)			
No	341(99.4)	398(98.5)	
Yes	2(0.6)	6(1.5)	0.25

Table 3 presents the unadjusted and adjusted effects of time (2010 vs. 2004) on odds of HIV and syphilis infection and behavioral characteristics. After adjusting for socio-demographics, FSWs enrolled in 2010 had 69% higher odds of collecting at least $16.30 USD per sexual act (Adjusted odds ratio [AOR]: 1.69, 95% CI: 1.17–2.43), 88% lower odds of having a primary sex partner in the last month (AOR: 0.12, 95% CI: 0.07–0.21), 86% lower odds of having unprotected sex with a client in the last month (AOR: 0.14, 95% CI: 0.09–0.21), 37% higher odds of having ten or more new sex partners in the last month (AOR: 1.37, 95% CI: 0.98–1.90), and 79% lower odds of reporting drug use in the last three months (AOR: 0.21, 95% CI: 0.08–0.52). Between 2004 and 2010, odds of having received an HIV test increased significantly by over 2.5 fold (AOR: 2.59, 95% CI: 1.79–3.73), while odds of HIV infection increased by four-fold, though not significantly (AOR: 4.12, 95% CI: 0.76–22.45). During this same period, odds of syphilis infection decreased by 35% and was of borderline statistical significance (AOR: 0.65, 95% CI: 0.41–1.03).

**Table 3:** Unadjusted and adjusted effects of time (2010 vs. 2004) on odds of HIV and syphilis infection and behavioral risk factors (n = 747).

Factors	OR, 2010/2004 (95% CI)	P-value	AOR, 2010/2004 (95% CI)	P-value
Average charge per sex trade≥16.3 US dollars	1.79 (1.29–2.47)	<0.001	1.69 (1.17–2.43)	0.01
Age at sex debut ≥18 years	0.98 (0.73–1.32)	0.89	0.98 (0.68–1.41)	0.90
Age of initiating sex work ≥21 years	1.36 (1.02–1.82)	0.04	1.14 (0.71–1.82)	0.59
Duration of sex work ≥2 years	1.40 (1.04–1.87)	0.03	1.01 (0.71–1.43)	0.96
Having primary sex partner(s) in the last 6 months	0.56 (0.42–0.76)	<0.001	0.12 (0.07–0.21)	<0.0001
Having unprotected sex with primary sex partner(s) in the last month	1.25 (0.87–1.80)	0.23	0.96 (0.61–1.50)	0.85
Number of sex trades in the last month ≥10	1.57 (1.12–2.19)	0.01	1.30 (0.90–1.88)	0.17
Having unprotected sex with clients in the last month	0.13(0.89∼0.19)	<0.0001	0.14(0.09∼0.21)	<0.0001
Number of new sex partner(s) in the last 1 months ≥10	1.51 (1.12–2.04)	0.01	1.37 (0.98–1.90)	0.07
Used drug in the last 3 months	0.23 (0.10–0.55)	<0.001	0.21 (0.08–0.52)	<0.001
Utilized MMT in the last 3 months	-	0.93	-	0.94
Received HIV testing	3.21 (2.31–4.47)	<0.0001	2.59 (1.79–3.73)	<0.0001
Syphilis (+)	0.74 (0.49–1.12)	0.15	0.65 (0.41–1.03)	0.07
HIV (+)	2.57 (0.52–12.82)	0.25	4.12 (0.76–22.45)	0.10

Notes: OR, odds ratio; CI, confidence interval; AOR, adjusted odds ratio, adjusted factors included age, ethnicity, previous occupation, currently married or cohabiting, yearly income, and birth area.

## Discussion

Harm reduction programs have been implemented in China since 2004 [18]–[21], [25], but there have been few data on HIV, syphilis infection and associated factors among FSWs after implementation of harm reduction programs. Based on two cross-sectional surveys conducted among FSWs before and after the implementation of harm reduction programs, the current study yielded encouraging, albeit mixed results.

Data from this study suggests that harm reduction strategies may be an effective means of reducing unprotected sex with clients, increasing HIV testing, and reducing risk of syphilis infection among FSWs in China. Six years after the introduction of multifaceted harm reduction programs in 2004, odds of syphilis infection and unprotected sex with clients decreased, while odds of HIV testing dramatically increased, even after taking into account sociodemographic discrepancies between 2004 and 2010 survey participants. Conceivably, lower odds of syphilis infection in 2010 may have been attributed to the higher rates of condom use with clients.

It remains unclear why odds of syphilis but not HIV infection declined between 2004 and 2010, but given the persistence of FSW's unprotected sex with primary partners and presuming that HIV prevalence among IDUs increased between 2004 and 2010, it is possible that some of the six HIV-positive participants in 2010 were infected via unprotected sex with their primary sex partners, who themselves contracted the HIV virus through injection drug use. It is worth noting that while there was no evidence from this study to indicate that harm reduction programs lowered odds of HIV infection among FSW in Xichang, there was likewise no evidence to rule out the possibility that such programs precluded a significant increase in the odds of HIV infection among FSWs. Moreover, if some FSWs were indeed infected with HIV by their primary partner, the harm reduction programs may in fact have prevented transmission of HIV from FSW to clients, as well as vice versa. Additional qualitative studies and sociocultural measurements are needed to contextualize HIV transmission and acquisition within FSWs' broader social environment.

In addition to persistent unprotected sex with primary sex partners before and after the introduction of harm reduction programs, odds of having more ten or more sex partners in the last month actually increased after harm reduction programs were implemented. We were unable to determine what may have caused the rise in number of sex partners, but it may have been partly attributed to local police crack downs on FSWs [6], that may have in turn disrupted the economics of commercial sex in Xichang. Conceivably, the 26.8% drop in total estimated number of sex workers in Xichang between 2004 and 2010 may have amplified the client-to-FSW ratio in Xichang city, whereby each FSW had a higher per-capita pool of potential clients. The fact that participants in 2010 reported higher remuneration per sexual act also lends circumstantial support to the theory that the ratio of client-demand to FSW-supply may have increased between 2004 and 2010. To the extent possible, leadership for future harm reduction initiatives targeting FSWs should consider collaborating with law enforcement institutions and accounting for the legal and socioeconomic environments of commercial sex work.

Despite the fact that HIV prevalence did not significantly increase from 2004 to 2010, it was disconcerting to find that HIV prevalence among Xichang FSWs in 2010 was notably higher than China overall. A systematic literature review of HIV and syphilis prevalence for FSWs in China showed relatively low HIV prevalence (median = 0.6%) [17], almost one-third the HIV prevalence among FSWs in Xichang city in 2010 (1.5%).

To be sure, Xichang's HIV epidemic has become one of the worst in China, with 2268 cumulative reported HIV/AIDS cases reported by the end of 2012. Part of the challenge of addressing HIV in Xichang is an epidemiological synergy and crossover between FSWs and injection drug use networks, as strongly suggested by their similar HIV molecular subtypes [27]. The fact that 58% of FSWs in the 2010 survey had engaged in sex work for less than two years underscores the transiency and high “turn-over” of sex work. Hence, women who use injection drugs and intermittently engage in sex work from time to time may be acting as another bridge in HIV transmission from IDU to FSW clients and the general population [23]–[25], [27].

Syphilis and the genital ulcers often associated with its infection is also another factor that is likely exacerbating the risk of HIV acquisition and transmission among FSWs in Xichang. Among FSWs, prevalence of syphilis infection in our 2010 survey was almost twice as high as the already troubling national median prevalence of 6.9% [17]. Nationwide, the annual number of national reported syphilis cases increased from 100,102 in 2004 to 448,620 in 2012 [15]. Effective sexual intervention programs to prevent HIV and syphilis transmission are urgently needed [26].

Findings from the study should be interpreted in light of several limitations. First, insufficient statistical power because of small sample sizes may have precluded detection of significant changes in HIV infection over time. Second, although controlled for in multivariate modeling, differences in age, ethnicity, previous occupation, currently married or cohabiting, yearly income and birth area between the 2004 and 2010 cross-sectional study suggested the possibility of selection bias that may have led to overestimation of the intervention's true impact. Third, ostensible effects of the harm reduction programs may in fact have been more attributed to unmeasured secular changes such as attitudes towards condoms.

Our previous study among Xichang IDUs found that sero-incidence of syphilis infection was high before and after implementation of IDU harm reduction programs, and that no significant changes over time were detected [25]. Arguably, the most troubling finding from the present study is the high prevalence of syphilis among FSW, considering that Xichang is located along a major drug trafficking route. High prevalence of syphilis which can cause genital ulcers could put FSWs and IDUs at a high risk of acquiring and transmitting HIV via sexual contacts [28]–[30]. The conditions for epidemiological synergy exist, which may suggest a bridging role of FSWs in HIV transmission from IDUs to general population. This study underscores the need for scientific approach to urgently needed to target to both of FSWs and IDUs and break their bridging networks for HIV transmission.
